# Which Boys and Which Girls Are Falling Behind? Linking Adolescents’ Gender Role Profiles to Motivation, Engagement, and Achievement

**DOI:** 10.1007/s10964-020-01293-z

**Published:** 2020-07-31

**Authors:** Junlin Yu, Ros McLellan, Liz Winter

**Affiliations:** Faculty of Education, University of Cambridge, Cambridge, UK

**Keywords:** Masculinity, Femininity, Gender roles, Latent profile analysis, Motivation, Academic achievement

## Abstract

Research on gender gaps in school tends to focus on average gender differences in academic outcomes, such as motivation, engagement, and achievement. The current study moved beyond a binary perspective to unpack the variations within gender. It identified distinct groups of adolescents based on their patterns of conformity to different gender norms and compared group differences in motivation, engagement, and achievement. Data were collected from 597 English students (aged 14–16 years, 49% girls) on their conformity to traditional masculine and feminine norms, growth mindset, perseverance, self-handicapping, and their English and mathematics performance at the end of secondary school. Latent profile analysis identified seven groups of adolescents (resister boys, cool guys, tough guys, relational girls, modern girls, tomboys, wild girls) and revealed the prevalence of each profile. Within-gender variations show that two thirds of the boys were motivated, engaged, and performed well in school. In contrast, half of the girls showed maladaptive patterns of motivation, engagement, and achievement, and could be considered academically at risk. By shifting the focus from “boys versus girls” to “which boys and which girls”, this study reveals the invisibility of well-performing boys and underachieving girls in educational gender gap research.

## Introduction

Boys lag behind girls in school across many Western industrialized countries (OECD 2015). On average, not only do boys report poorer quality motivation (Butler 2014), they also tend to be less engaged (Lam et al. 2012) and perform worse than girls in secondary school (Voyer and Voyer 2014). However, research on binary gender differences risks treating boys and girls as two homogenous groups, masking considerable variations in motivation, engagement, and achievement within each gender. To unpack within-gender heterogeneity, some studies examine the extent to which adolescents conform to traditional gender roles and reveal that rigid conformity to traditional gender roles is associated with lower academic motivation (C. S. Brown 2019), engagement (Ueno and McWilliams 2010), and achievement (Santos et al. 2013). Although this is an important step forward, adolescents within each gender group may vary not only in their *degree* of gender role conformity, but also in their *patterns* of conformity to different gender roles. Distinct patterns of gender role conformity may in turn differentially predict students’ academic outcomes. The current study aimed to identify subgroups of adolescent boys and girls based on their emergent patterns of gender role conformity, and compare group differences in motivation, engagement, and achievement in English and mathematics. By shifting the focus from “boys versus girls” to “which boys and which girls”, the current study can provide a fresh look at the extent of boys’ problems in education and draw attention to underachieving girls in school.

### Gender Role Conformity and Implications for Academic Success

Gender roles are widely shared beliefs about what constitutes gender-appropriate behaviors in a given society at a given time (Wood and Eagly 2012). The study of gender role conformity concerns the extent to which individuals conform to normative expectations of how to be a “real” man or woman. Although adolescents may express less rigid views about what men and women in general should do, many of them conform to traditional gender roles in their personal lives (Whitehead 2003). The present study thus focused on the impact of adolescents’ gender role conformity on their school success.

Although many aspects of traditional gender norms have been identified, the current study focused on nine central tenets of masculinity and femininity in Western cultures. Five of the norms reflect traditional masculinity: emotional control, competitiveness, aggression, self-reliance, and risk-taking; and four of them reflect traditional femininity: thinness, appearance orientation, romantic relationships, and housekeeping or domestic duties. These norms are selected because (a) they represent recurring themes in qualitative studies on meanings of masculinity and femininity (e.g., Holland and Eisenhart 1990; Munsch and Gruys 2018), (b) are deemed important and assessed across multiple commonly used measures of gender norms (Levant et al. 1992; Mahalik et al. 2003), and (c) continue to be highly relevant for contemporary construction of masculinity and femininity (Parent et al. 2020). As a result, they may reflect prevailing gender norms that influence large segments of the population. Although moderate expressions of these norms can be positive (e.g., being independent or being well-groomed), strict adherence to these norms may become prohibitive when they lead to rigidly gendered thoughts, feelings, behaviors, and interests (e.g., help avoidance or preoccupation with appearance).

Indeed, individuals’ degree of gender role conformity matters, and rigid constructions of masculinity can undermine boys’ and young men’s academic success. For example, male students who feel pressured to appear emotionally detached and self-reliant have been found to adopt a surface approach to learning (Marrs 2016), avoid seeking help in the classroom (Leaper et al. 2019), report lower levels of school engagement (A. A. Rogers, DeLay, et al. 2017), and perform worse academically (Santos et al. 2013). Boys who display high levels of physical aggression, competitiveness, and risky behaviors may experience more interpersonal conflict with their teachers and peers, thereby reducing their odds of success in school (Ueno and McWilliams 2010). Male students who endorse the physical aggression norm also report lower levels of mastery goals (Marrs 2016) and school enjoyment (Rogers et al. 2017). Meanwhile, by conceptualizing conformity as ranging along a continuum, prior studies indicate that boys and young men who reject rigid conformity to traditional masculine norms tend to be more academically successful.

Similarly, strict adherence to traditional notions of femininity can hinder girls’ and young women’s academic success. Adolescent girls who are preoccupied with their appearance and body image tend to report lower academic self-efficacy, fewer mastery goals (Brown 2019), greater skepticism toward school (Nelson and Brown 2019), and show lower effort and academic performance (McKenney and Bigler 2016). In contrast, those who reject these restrictive feminine norms tend to show higher levels of motivation and performance. Additionally, young women who indulge in romantic fantasies tend to report lower educational goals and less interest in male-typed domains such as mathematics and science (Park et al. 2011). Adolescent girls who expect to take up the homemaker role have also been found to perform worse academically (Whitehead 1994).

Studies reviewed above show that young people’s conformity to their own gender’s norms can shape their motivation, engagement, and achievement. However, the expression of masculinity or femininity is not restricted to a single gender. Many preadolescent girls self-identify as tomboys and enact stereotypically masculine behaviors (Paechter 2010), and some teenage boys attend to their appearance to maintain a cool “laddish” image (Jackson 2006a). In addition, conforming to traditional masculine/feminine norms appears to influence boys and girls in similar ways. Adolescent girls who adhere to masculine norms such as restrictive emotionality and physical aggression show lower levels of behavioral self-regulation (Liang et al. 2019) and school belonging (Huyge et al. 2015), and young men who possess more romantic fantasies report lower educational aspirations (Rudman and Heppen 2003). The present study, therefore, examined adolescents’ adherence to both their own gender’s and the other gender’s norms to understand the complex patterns and implications of gender role conformity among contemporary youth.

### Patterns of Gender Role Conformity

Studies discussed earlier show that rigid adherence to traditional gender norms can undermine students’ learning and achievement. However, people may adhere to multiple facets of traditional gender norms simultaneously and to varying degrees, which produces different patterns of gender role conformity. For instance, one study used cluster analysis to identify subgroups of female undergraduate students based on their orientations toward thinness, romance, perfectionism, self-objectification, and contingent self-worth (Schrick et al. 2012). Four distinct profiles were found, ranging from a group of “Other-Focused” women who strongly endorsed thinness, perfectionism, and self-objectification, to a group who rejected the thinness norm and also scored low on the other dimensions. In addition, Other-Focused women had the lowest level of academic engagement and the highest psychological distress, whereas women who rejected the thin ideal showed the highest academic engagement and the lowest distress. These findings illustrate the utility of adopting a pattern perspective to understand how conformity or resistance to multiple gender norms can work in tandem to influence students’ academic success.

The current study aimed to quantitatively identify subgroups of adolescent boys and girls based on their conformity to a range of masculine and feminine norms. This approach has conceptual parallels to ethnographic studies that identify subgroups of boys and girls based on their “doing of gender” (West and Zimmerman 1987). This line of qualitative inquiry has consistently identified a group of schoolboys who conform to conventional ideals of masculinity, labeled as “lads” in the UK (Jackson 2006a) or “jocks” in the US (Pascoe 2003). Similarly, several images of schoolgirls have been identified, ranging from “tomboys” who reject conventional femininity (Paechter 2010), to “wild girls” who enact stereotypically masculine behaviors—such as being loud, disruptive, and aggressive—while wearing tight and revealing clothing to emphasize their physical attractiveness (Jackson 2006b). Although this body of work provides a nuanced understanding of multiple masculinities and femininities in school, these typologies are often based on small samples in a particular setting, and it remains unclear whether they represent common ways for boys and girls to “do gender” in school. In contrast, the current study aimed to identify emergent gender role profiles in a large sample of adolescents across multiple schools and examine the prevalence of each profile. In doing so, it can provide critical information about which gender role profiles typically emerge during adolescence and are therefore meaningful to study in research.

### Motivation, Engagement, and Achievement

To understand the impact of gender role conformity on school success, it is crucial to examine the link between different gender role profiles and students’ achievement. Importantly, the image of a school subject can powerfully shape students’ achievement, depending on whether doing well in that subject is seen as compatible with one’s gender role (Kessels et al. 2014). For example, girls’ conformity to traditional masculinity has been associated with better performance in a male-typed subject such as mathematics, but poorer performance in a female-typed subject such as English (Leaper et al. 2019). The current study thus examined students’ performance in both English and mathematics to fully gauge the impact of gender role conformity on academic achievement.

Beyond achievement outcomes, it is important to investigate students’ motivation and engagement, which are both influenced by gender role beliefs and can influence subsequent performance (Wigfield et al. 2015). One influential approach to the study of motivation is the mindset theory, which centers on students’ beliefs or mindsets about ability (Dweck and Molden 2017). A growth mindset refers to the belief that one’s ability is malleable and can be developed through effort. This is in contrast to a fixed mindset, or the belief that one’s ability is mostly innate and cannot be changed. A consistent finding in educational research is that having a growth mindset is linked to positive beliefs about effort, stronger mastery goals, less low-ability attributions for failure (Dweck and Molden 2017), and greater intrinsic motivation (Dinger et al. 2013). Therefore, mindset can provide insights into students’ broad system of motivation. In addition, school engagement has been conceptualized as encompassing cognitive, emotional, and behavioral dimensions (Fredricks et al. 2004). This study focused on two behavioral aspects of engagement, namely perseverance and self-handicapping, as behavioral engagement is closely related to students’ achievement. Perseverance reflects positive behaviors in learning and is linked to a growth mindset and higher achievement (Burnette et al. 2013). Self-handicapping, on the other hand, involves deliberately withholding effort to create face-saving excuses for potential poor performance. It reflects problematic learning behaviors and is associated with a fixed mindset (Rhodewalt 1994) and lower achievement (Schwinger et al. 2014).

An understanding of students’ mindset, perseverance, and self-handicapping may reveal key processes contributing to some boys’ underachievement. Qualitative studies of adolescent boys in the UK (Jackson 2002) and young men in the US (Munsch and Gruys 2018) found that many male students aspired to “effortless achievement” and considered it central to the construction of masculinity. They espoused the belief that achievement without effort signaled natural intelligence, and that failure without trying could be attributed to a lack of effort rather than a lack of ability. Based on their beliefs about effort and attributional style, it is plausible that boys who conform to traditional masculinity might perceive ability as fixed, view effortful persistence as an indication of low ability, and withhold effort to avoid the implications of failure. These maladaptive beliefs and behaviors may, in turn, undermine boys’ achievement.

Similarly, these constructs may provide insights into why some girls perform less well in school, especially in male-typed subjects. Girls who adhere strongly to traditional femininity may be more susceptible to the gender stereotype that they lack the fixed innate talent to succeed in mathematics (Leslie et al. 2015). Furthermore, despite the general perception of girls as diligent students, a three-year longitudinal study revealed a steady increase of disengagement among adolescent girls. Specifically, girls reported a greater tendency to give up and self-handicap in schoolwork after the transition to secondary school (Burns et al. 2019). These findings suggest that examining perseverance and self-handicapping has the potential to capture the quiet disengagement among girls that might otherwise go unnoticed by their teachers.

## Current Study

The present study transcended the traditional gender binary to examine which boys and which girls were falling behind in school. Specifically, it addressed two research questions. First, what are the emergent gender role profiles during adolescence and how common are these profiles? The current study used latent profile analysis to identify adolescents with similar patterns of conformity across nine salient aspects of traditional gender norms. Since no studies to our knowledge have created profiles based on adolescents’ simultaneous adherence to a range of masculine and feminine norms, it was difficult to predict what profiles would emerge. Nevertheless, it was reasonable to expect that some emergent profiles might match various images of boys and girls already documented in qualitative studies (e.g., jocks, tomboys).

Second, how do the emergent gender role profiles relate to students’ motivation, engagement, and achievement? The current study examined the cross-sectional associations between gender role profiles and students’ mindset, perseverance, and self-handicapping, as well as the longitudinal associations between gender role profiles and students’ achievement in English and mathematics. Since rigid conformity to traditional masculinity and femininity has been negatively associated with school success for boys and girls alike, profiles endorsing multiple aspects of traditional gender norms were expected to be less academically successful. In contrast, profiles showing resistance to rigid constructions of gender were expected to display more adaptive motivation and engagement, as well as better academic performance.

## Methods

### Participants and Procedure

The sample consisted of 597 students from four state secondary schools in England (291 girls, aged 14-16 years). Participants were in the last two years of compulsory education (Year 10: *n* = 395, Year 11: *n* = 202) and were working toward the national high-stakes General Certificate of Secondary Education (GCSE) exams taken at the end of Year 11. The average level of student achievement was diverse across schools: the proportion of students obtaining a pass grade in GCSE English and mathematics ranged from 42-74% in each school. The majority of participants self-identified as White (83%), with the remaining students identifying as Black (6%), Asian (5%), or mixed race or other (6%). Thirteen percent of students indicated that they had been eligible for free school meals at some point within the last 6 years—an indicator of low family income.

The study was reviewed and approved by the departmental ethics committee. Before data collection, parents were informed of the study and given the opportunity to withdraw their child. Questionnaires assessing gender role conformity, motivation, and engagement were group administered to students during regular school hours and took approximately 20 minutes to complete. Teachers responsible for administering the questionnaire were provided with an instruction sheet containing the purpose, ethics, and procedures of the study. Students were told that their participation was completely voluntary and that no one at home or school would see their answers. Participants subsequently took the GCSE exams at the end of Year 11, and their achieved grades in English and mathematics were obtained directly from schools. The time lag between self-report measures and achievement outcomes was introduced to understand the association between students’ patterns of gender role conformity and their subsequent academic performance.

### Measures

The questionnaire contained three sections: conformity to traditional gender roles (48 items), motivation and engagement in English (13 items), and motivation and engagement in mathematics (13 items). The order of the sections was counterbalanced and the items within each section were randomized.

#### Gender role conformity

Students’ conformity to traditional masculinity was assessed by five subscales from the Conformity to Masculine Norms Inventory-46 (Parent and Moradi 2009): Emotional Control (“I tend to keep my feelings to myself”), Winning (“In general, I will do anything to win”), Violence (“Sometimes violent action is necessary”), Self-reliance (“It bothers me when I have to ask for help”), and Risk-taking (“I frequently put myself in risky situations”). Conformity to traditional femininity was measured by four subscales: Thinness (“I am always trying to lose weight”), Appearance Orientation (“I check my appearance in a mirror whenever I can”), Romantic Relationship (“Being in a romantic relationship is important”), and Domestic (“I enjoy spending time making my living space look nice”). The Appearance Orientation subscale was adapted from the Multidimensional Body-Self Relations Questionnaire (Brown et al. 1990), and the other three subscales were taken from the Conformity to Feminine Norms Inventory-45 (Parent and Moradi 2010). All statements were phrased in the first person to assess participants’ personal conformity to traditional gender roles. Consistent with the original conceptualization (Mahalik et al. 2003), items were rated on a 4-point scale (0 = *Disagree strongly*, 3 = *Agree strongly*) to capture extreme nonconformity to extreme conformity. Appropriate items were reverse scored so that higher scores represented greater conformity to a given aspect of the traditional gender norm.

#### Motivation

Students’ growth mindset was measured to capture their broad motivational orientation. Students’ mindset was assessed by a brief 3-item scale (De Castella and Byrne 2015). Statements reflected a fixed mindset and students’ responses were reverse scored so that higher scores corresponded to a stronger growth mindset (“To be honest, I don’t think I can really change how good I am at …”). These items were rated on a 6-point scale ranging from 1 (*Disagree a lot*) to 6 (*Agree a lot*).

#### Engagement

Students’ behavioral engagement was assessed via perseverance and self-handicapping. Perseverance reflects positive behaviors in learning and is defined as the extent to which students maintain effort when encountering challenges. Perseverance was assessed with a 4-item scale (Elliot et al. 1999). An example item is “If a particular topic or problem confuses me in my … lesson, I go back and try to figure it out”. Self-handicapping, on the other hand, reflects problematic learning behaviors and refers to intentional effort withdrawal to create excuses for potentially poor performance. Students reported the frequency of self-handicapping behavior on a 6-item scale adapted from the Patterns of Adaptive Learning Scale (Midgley et al. 2000). An example item is “Sometimes I purposely get involved in lots of activities. Then if I don’t do so well in … as I hoped, I can say it is because I was too involved in other things.” These items were rated on a 6-point scale ranging from 1 (*Disagree a lot*) to 6 (*Agree a lot*).

#### Achievement

Academic achievement was operationalized as English and mathematics performance in the national GCSE exams at the end of secondary school. Grades ranged from 1 (the lowest) to 9 (the highest) and were standardized before analyses to ease interpretation. Since the current study focused on the independent effect of gender role profiles on students’ performance beyond prior achievement, students’ English and mathematics grades on National Curriculum Tests were also gathered as indicators of prior achievement and were included as a covariate in analyses. These tests are taken by all students in England at the end of primary school and represent the only national test data available prior to GCSE.

### Analytic Strategy

Data analysis proceeded in three steps. Measurement invariance and factor structure of the scales were first evaluated using exploratory structural equation modeling (ESEM; Asparouhov and Muthén 2009). Next, factor scores saved from the best fitting measurement models were used to conduct latent profile analyses. Once the optimal profile solution was determined, differences in motivation, engagement, and achievement were compared across profiles. All latent variable analyses were conducted in Mplus 8.3 (Muthén and Muthén 1998-2017).

#### Measurement models

Simulation studies show that it is inappropriate to treat ordinal scales with fewer than five categories as continuous variables (Rhemtulla et al. 2012). Consequently, the gender role measures were modeled as categorical variables using the weighted least square estimator (WLSMV), and the mindset and engagement measures as continuous variables using the robust maximum likelihood estimator (MLR).

Since measures of masculinity had been primarily validated among males and measures of femininity among females, the present study first examined the invariance of the gender role measures to ensure that salient dimensions of masculinity and femininity had the same meaning to boys and girls. Four levels of invariance were tested: configural, weak, strong, and strict (Gregorich 2006). Multigroup ESEMs were initially estimated to test whether the factorial structural was the same across gender (configural invariance). Equality constraints were then added to the factor loadings (weak invariance), thresholds (strong invariance), and residual variances (strict invariance). Each level of invariance was established if the more restricted model did not show a significant deterioration in fit compared to the previous model. According to Chen (2007), weak invariance is supported if ΔCFI < 0.010, ΔRMSEA < 0.015, and ΔSRMR < 0.030. Strong or strict invariance is supported if ΔCFI < 0.010, ΔRMSEA < 0.015, and ΔSRMR < 0.010. Factor scores from the most invariant model were saved as input for latent profile analyses.

Next, the factor structure of the mindset and engagement scales was verified in ESEM models. Items were specified to load on their respective factors and cross-loadings were targeted to be as close to zero as possible using target rotation. Model fit was assessed using the comparative fit index (CFI), the root mean square error of approximation (RMSEA), and the standardized root mean-square residual (SRMR). Good model fit was indicated by a CFI value close to 0.95 or above, RMSEA close to .06 or below, and SRMR close to 0.08 or below (Hu and Bentler 1999). Factor scores (*M* = 0, *SD* = 1) from the ESEM models were saved and used as outcomes of latent profile membership.

#### Latent profile analyses

Models with up to six profiles were computed for boys and girls separately to identify subgroups of adolescents who showed similar patterns of gender role adherence. The optimal number of profiles to retain was guided by several criteria (Nylund et al. 2007). First, the Akaike information criteria (AIC), the consistent AIC (CAIC), the Bayesian information criteria (BIC), and sample-size adjusted BIC (SABIC) were used to assess the model fit, with lower values suggesting a better fitting model. These indices were plotted in a scree-like plot to identify the elbow point after which adding additional profiles led to minimal gains in model fit. Additionally, the bootstrap likelihood ratio test (BLRT) was computed for each solution, and a non-significant BLRT test supports a model with one less profile. The theoretical interpretability of the profiles was also considered. Lastly, the entropy value (ranging from 0 to 1) was used as an indicator of classification accuracy, with higher values representing greater precision in classification.

#### Outcomes of latent profile membership

Differences in academic outcomes across profiles were compared using the BCH method (Asparouhov and Muthén 2014), which is conceptually equivalent to a weighted ANOVA. To test the cross-sectional associations between gender role profiles and students’ motivation and engagement, a default version of this method was performed in Mplus. To examine the longitudinal associations between gender role profiles and students’ English and mathematics performance, a manual BCH was performed to allow for the inclusion of prior achievement as a covariate. Profile-specific means were then compared to test whether gender role profiles had an independent effect on students’ academic performance after accounting for prior achievement.

## Results

### Preliminary Analyses

Missing values for the items were minimal (ranging from 0.2-4%) and were imputed using the expectation-maximization algorithm in SPSS. Multigroup-ESEM models supported the strict invariance of the gender role measures, as all changes in CFI, RMSEA, and SRMR fell within acceptable ranges (see Supplementary Appendix A). This suggests that measures of traditional masculinity and femininity carried the same meaning and functioned equivalently for boys and girls. All items loaded highly on the target factors (Emotional control: 0.61 to 0.82; Winning: 0.64 to 0.87; Violence: 0.51 to 0.77; Self-reliance: 0.61 to 0.74; Risk-taking: 0.62 to 0.89; Thinness: 0.60 to 0.91; Appearance: 0.56 to 0.81; Romance: 0.42 to 0.86; Domestic: 0.73 to 0.83) and much more weakly on other factors (below 0.30). In addition, a 6-factor ESEM model showed that most items assessing mindset, perseverance, and self-handicapping in English and mathematics loaded highly on their respective factors (ranging from 0.44 to 0.90). One perseverance item and two self-handicapping items in mathematics showed cross-loadings on the corresponding construct in English, but these cross-loadings were always smaller than the target loadings, and the overall fit of the measurement model was excellent (CFI = 0.966, RMSEA = 0.039, SRMR = 0.022). No residual covariances were specified in any measurement model.

Means, standard deviations, and reliability estimates for all variables are presented in Table 1. Average gender differences were found for the majority of gender role measures, suggesting that they captured normative standards of masculinity and femininity. Boys conformed more strongly to masculine norms such as winning, violence, and risk-taking, whereas girls adhered more strongly to feminine norms such as thinness, appearance orientation, and domesticity. Although the current study focused on variations in academic outcomes within gender, mean gender differences consistent with prior studies were also observed. On average, girls reported greater perseverance and performed better in English, whereas boys endorsed a stronger growth mindset and earned better grades in mathematics.

**Table 1:** Descriptive statistics and reliability estimates for variables

Variable	Cronbach’s *α*	Girls Mean (SD)	Boys Mean (SD)	Cohen’s *d*
Emotional control	0.87	1.72 (0.73)	1.72 (0.67)	0.01
Winning	0.90	1.35 (0.75)	1.62 (0.70)	−0.38***
Violence	0.82	1.54 (0.64)	1.89 (0.61)	−0.55***
Self-reliance	0.84	1.28 (0.74)	1.17 (0.64)	0.15
Risk-taking	0.86	1.25 (0.67)	1.50 (0.70)	−0.36***
Thinness	0.88	1.62 (0.86)	1.03 (0.71)	0.75***
Appearance orientation	0.82	1.90 (0.60)	1.37 (0.70)	0.81***
Romantic relationship	0.71	1.33 (0.61)	1.43 (0.59)	−0.16
Domestic	0.86	1.99 (0.72)	1.73 (0.70)	0.36***
English mindset	0.82	4.23 (1.09)	4.21 (1.17)	0.02
English perseverance	0.79	4.11 (1.00)	3.88 (0.92)	0.24**
English self-handicapping	0.86	2.09 (0.91)	2.14 (0.92)	−0.05
English achievement	/	5.96 (1.56)	5.28 (1.68)	0.42***
Math mindset	0.80	4.22 (4.16)	4.58 (1.03)	−0.32***
Math perseverance	0.81	4.16 (1.01)	4.27 (0.92)	−0.12
Math self-handicapping	0.83	2.18 (0.90)	2.07 (0.93)	0.12
Math achievement	/	5.48 (1.78)	5.93 (1.99)	−0.24**

*Note*. Positive Cohen’s *d* values indicate higher scores for girls

*p < 0.05, **p < 0.01, ***p < 0.001

Intercorrelations among the observed variables (Table 2) show that when significant correlations were found, conformity to most traditional gender norms was associated with a weaker growth mindset, reduced perseverance, and increased self-handicapping. The only exception was the Domestic subscale, which was related positively to perseverance and negatively to self-handicapping for boys and girls alike. A closer look at the item content suggests that the items might be tapping into orderliness and organization (e.g., “I enjoy spending time making my living space look nice”). Regarding academic-related measures, across both subjects, growth mindset and perseverance were associated with better academic achievement, whereas self-handicapping was associated with worse achievement.

**Table 2:** Intercorrelations among variables for boys (below the diagonal) and girls (above the diagonal)

	Variable	1	2	3	4	5	6	7	8	9	10	11	12	13	14	15	16	17
1	Emotional control		**0.15**	**0.21**	**0.56**	**0.23**	**0.17**	0.00	**−0.15**	−0.07	**−0.15**	**−0.12**	0.06	−0.07	**−0.19**	**−0.17**	0.08	−0.07
2	Winning	**0.17**		**0.27**	0.10	**0.22**	0.06	0.08	−0.01	−0.06	−0.11	−0.07	0.03	0.06	0.00	−0.08	−0.06	0.05
3	Violence	**0.28**	**0.30**		**0.17**	**0.33**	0.11	0.05	−0.02	**−0.29**	**−0.16**	**−0.31**	**0.16**	−0.05	−0.11	**−0.37**	**0.20**	−0.06
4	Self-reliance	**0.44**	**0.18**	**0.14**		**0.20**	**0.18**	−0.03	0.03	**−0.18**	**−0.23**	**−0.22**	**0.22**	−0.03	**−0.30**	**−0.30**	**0.21**	−0.03
5	Risk-taking	**0.22**	**0.33**	**0.35**	**0.16**		**0.15**	0.04	−0.07	**−0.22**	−0.05	−0.11	**0.21**	−0.06	−0.05	**−0.19**	**0.18**	−0.07
6	Thinness	0.07	−0.03	0.06	0.11	−0.02		**0.34**	**0.27**	−0.09	−0.06	−0.03	0.11	0.02	−0.05	−0.11	0.09	−0.08
7	Appearance orientation	**−0.13**	**0.23**	0.06	−0.02	**0.12**	**0.20**		**0.35**	**0.19**	0.00	0.01	0.11	0.03	0.02	0.00	0.09	**−0.13**
8	Romantic relationship	**−0.20**	0.07	−0.05	−0.06	0.06	0.05	**0.31**		0.04	−0.02	−0.07	**0.18**	−0.07	0.00	−0.04	**0.22**	**−0.17**
9	Domestic	**−0.15**	0.01	**−0.16**	**−0.14**	−0.08	0.00	**0.20**	**0.17**		0.06	**0.18**	**−0.24**	−0.05	0.00	**0.16**	**−0.18**	**−0.19**
10	English mindset	**−0.13**	0.04	**−0.15**	**−0.12**	−0.09	**−0.15**	−0.07	−0.01	0.07		**0.38**	**−0.39**	**0.14**	**0.33**	**0.17**	**−0.19**	−0.04
11	English perseverance	**−0.23**	−0.08	**−0.27**	**−0.33**	**−0.19**	−0.06	0.07	0.02	**0.21**	**0.42**		**−0.46**	**0.18**	**0.13**	**0.46**	**−0.33**	−0.11
12	English self-handicapping	**0.14**	**0.17**	0.10	**0.30**	**0.27**	**0.17**	**0.14**	**0.19**	−0.11	**−0.31**	**−0.40**		**−0.15**	**−0.18**	**−0.33**	**0.67**	−0.03
13	English achievement	**−0.14**	0.04	−0.08	−.03	−**0.15**	−0**.13**	−0.08	−0.08	−0.02	**0.19**	**0.14**	−0**.26**		**0.17**	**0.17**	−0**.23**	**0.55**
14	Math mindset	−**0.13**	0.06	−0.03	−0**.23**	−0.08	−**0.17**	−0.07	−0.03	0.11	**0.34**	**0.21**	−0**.27**	**0.28**		**0.40**	−0**.34**	**0.27**
15	Math perseverance	−0**.18**	−0.07	−0**.15**	−0**.28**	−0.11	−0.07	−0.03	0.07	**0.21**	0.06	**0.41**	−0**.35**	**0.28**	**0.39**		−0**.52**	**0.14**
16	Math self-handicapping	**0.18**	**0.17**	0.10	**0.31**	**0.23**	**0.14**	**0.18**	**0.15**	−0**.13**	−0**.17**	−0**.27**	**0.65**	−**0.30**	**−0.45**	−0**.55**		−0**.20**
17	Math achievement	−0.07	0.00	−0.03	0.00	−0.09	−0**.13**	−0**.21**	−0.06	−0**.13**	**0.14**	0.06	−0**.21**	**0.61**	**0.35**	**0.41**	−0**.39**	

*Note*. Significant correlations at the 0.05 level are shown in bold

### Gender Role Profiles Among Adolescent Boys and Girls

Fit indices for the 2- to 6-profile solutions among boys and girls can be found in Supplementary Appendix B. BLRT tests were significant for all the solutions and provided limited information to determine the optimal number of profiles. Changes in information criteria were also plotted to aid the model selection (see Supplementary Appendix C). These plots showed a clear inflection point at three profiles for both boys and girls. Inspection of the 3-profile solution among boys confirmed that these profiles were distinct and theoretically interpretable. Furthermore, the classification accuracy was high for the 3-profile solution (entropy = 0.81). Therefore, the 3-profile solution was retained as the final solution for boys.

Although the scree-like plot similarly pointed to a 3-profile solution among girls, entropy values indicated that the classification quality was suboptimal for the 3-profile model (entropy = 0.67), and that moving to a 4-profile solution resulted in a reasonably high entropy value (entropy = 0.76) as well as a high level of classification accuracy of participants to their most likely profile (ranging from 0.86 to 0.89; see Supplementary Appendix D). Comparing the 3- and 4-profile solutions indicated that the 3-profile solution combined two very different profiles into one, whereas the 4-profile solution allowed for the separation of the two (i.e., modern girls and wild girls; discussed below). Consequently, the 4-profile model was deemed superior to the 3-profile solution and was retained as the final solution for girls.

**Fig. 1:**
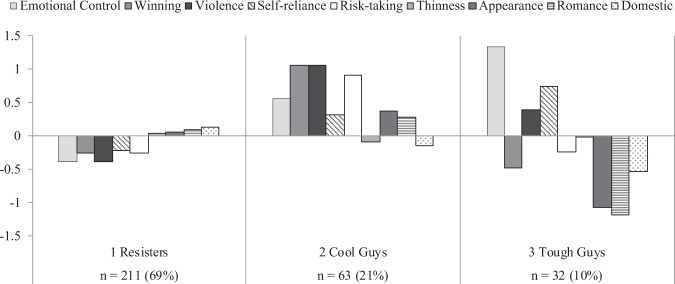
Patterns of gender role conformity among boys

**Fig. 2:**
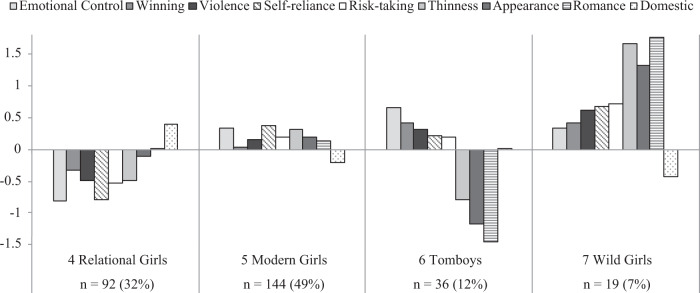
Patterns of gender role conformity among girls

Gender role profiles for boys and girls are graphically presented in Figs 1 and 2, and the means of each indicator for different profiles are reported in Supplementary Appendix E. The profiles were compared against different images of boys and girls in the literature, and existing labels were adopted whenever possible. Three groups of boys were identified—resisters, cool guys, and tough guys—each displaying a distinct pattern of gender role conformity. Profile 1 was the largest group of boys in this study (69%). Boys in this profile could be distinguished from all other boys by their resistance to traditional masculinity and ambivalence toward traditional femininity. Boys in Profile 2 were characterized by a macho and cool image. They strongly endorsed conventional ideals of masculinity, especially winning, violence, and risk-taking, while attaching importance to their appearance and romantic relationships. A fifth of the adolescent boys displayed this cool masculinity. Lastly, boys in Profile 3 portrayed an emotionally tough and “hard” image. Not only did they uphold the masculine norms of emotional stoicism, extreme self-reliance, and physical aggression, they were also the only group of boys who distanced themselves from stereotypically feminine qualities. This was the smallest profile and comprised only 10% of the boys.

Four groups of girls were identified, namely relational girls, modern girls, tomboys, and wild girls. Girls in Profile 4 (32%) were labeled as relational because they strongly rejected the norms of restrictive emotionality and extreme self-reliance. In other words, these girls were comfortable with connecting with others emotionally and asking others for help. Compared with other groups, relational girls also dismissed the majority of traditional gender norms, including the thin body ideal. Girls in Profile 5 embodied a hybrid version of femininity. They attached moderate importance to looking thin, attractive, and romantically desirable. Meanwhile, they endorsed the masculine norms of emotional control and extreme self-reliance. Put differently, these modern girls experienced discomfort in openly expressing feelings or seeking help from others. This group was the most prevalent profile and consisted of 49% of girls. Profile 6 (12%) corresponded to a group of boylike girls who are commonly thought of as tomboys: they were completely uninterested in traditional feminine qualities and enacted stereotypically masculine behaviors. Lastly, there was a small subset of girls who could be labeled as wild girls (7%). Similar to modern girls, wild girls embodied both masculine and feminine qualities but in a more extreme manner. They fully embraced traditional masculine norms while presenting themselves as romantically desirable and overtly feminine in appearance.

Although not the focus of the present study, additional analyses were performed to explore whether socioeconomic status (regular vs. free school meal eligibility) and race/ethnicity (White vs. non-White ethnic minorities) predicted profile membership (see Supplementary Appendix F). For boys, neither socioeconomic status nor race/ethnicity significantly predicted profile membership. For girls, those from low-income families had an increased likelihood of being classified as modern girls or tomboys (vs. relational girls). Girls from ethnic minority backgrounds had a greater likelihood of being classified as tomboys (vs. modern girls).

Based on adolescents’ patterns of gender role conformity, this study identified seven emergent subgroups of adolescents (resister boys, cool guys, tough guys, relational girls, modern girls, tomboys, wild girls) and revealed the prevalence as well as the socio-demographic composition of these profiles.

### Associations Between Gender Role Profiles and Academic Outcomes

**Fig. 3:**
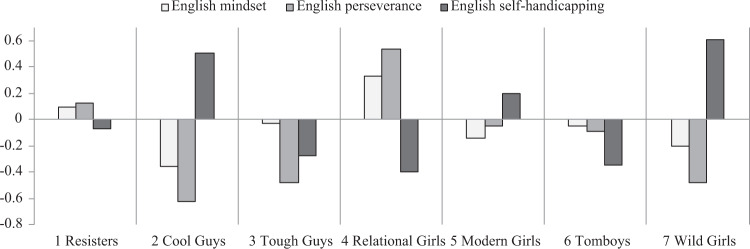
Patterns of English mindset, perseverance, and self-handicapping by profile

**Fig. 4:**
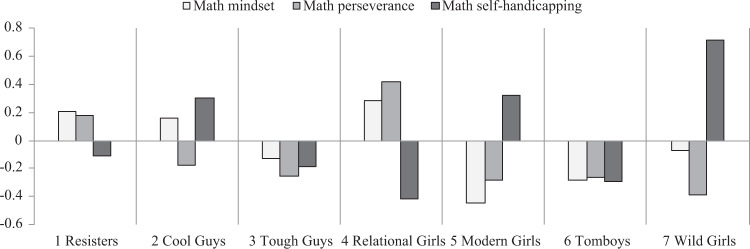
Patterns of mathematics mindset, perseverance, and self-handicapping by profile

The next aim was to examine whether students’ patterns of gender role conformity were associated with their concurrent motivation and engagement in English and mathematics. Figures 3 and 4 display the patterns of mindset, perseverance, and self-handicapping for the seven profiles, and the mean values of these outcomes are reported in Supplementary Appendix G. Consistent with the expectation, profiles resisting traditional gender norms were better academically adjusted than profiles conforming to these restrictive norms. Among the three groups of boys, resisters showed the most adaptive patterns of motivation and engagement. Compared with other boys, resisters consistently showed the highest levels of growth mindset and perseverance, as well as low levels of self-handicapping in English and mathematics. In contrast, cool guys showed arguably the least adaptive patterns of motivation and engagement. They reported low levels of perseverance and the highest levels of self-handicapping, especially in English. They were also the only group who held different mindsets for different subjects: they reported a fixed mindset in English but a growth mindset in mathematics. Tough guys displayed a somewhat mixed pattern of motivation and engagement. Across both subjects, they showed equally low levels of self-handicapping as resister boys. However, tough guys reported low levels of perseverance in learning, especially in English.

Among the four groups of girls, relational girls consistently displayed the most adaptive patterns of motivation and engagement. They reported the highest levels of growth mindset and perseverance, as well as the lowest levels of self-handicapping across both subjects. In contrast, wild girls and modern girls could be considered at risk academically. Compared with other girls, these two groups were characterized by low levels of growth mindset and perseverance, and high levels of self-handicapping, especially in mathematics. Tomboys showed a somewhat mixed pattern. Across both subjects, tomboys reported equally low levels of self-handicapping as relational girls, but were much less likely to hold a growth mindset or persist through challenges. This was particularly the case in mathematics.

Lastly, the longitudinal associations between students’ gender role profiles and their academic achievement at the end of secondary school were examined. Students’ prior achievement was included as a covariate to understand the independent effect of gender role profiles on students’ subsequent performance. Mean differences in English and mathematics achievement across different profiles are reported in Tables 3 and 4. Among the three groups of boys, tests of overall mean differences were significant for English achievement, *χ*^2^(2) = 14.29, *p* < 0.001, and marginally significant for mathematics achievement *χ*^2^(2) = 5.13, *p* = 0.08. Pairwise comparisons indicated that resister boys obtained the highest scores in English and mathematics, whereas cool guys obtained the lowest scores in both subjects. Tough guys achieved somewhat mixed results. Compared with cool guys, tough guys performed equally poorly in English but showed a trend toward better performance in mathematics.

**Table 3:** Mean differences in English and mathematics achievement across profiles of boys

Variable	1 Resisters	2 Cool Guys	3 Tough Guys
English achievement	−0.07a (0.06)	−0.56b (0.12)	−0.42b (0.17)
Mathematics achievement	0.15a (0.05)	−0.13b (0.10)	0.12ab (0.15)

*Note*. Means with different subscripts in the same row are significantly different at *p* < 0.05. Values in parentheses are standard errors

**Table 4:** Mean differences in English and mathematics achievement across profiles of girls

Variable	4 Relational Girls	5 Modern Girls	6 Tomboys	7 Wild Girls
English achievement	0.44a (0.10)	0.13b (0.08)	0.10b (0.16)	0.10b (0.14)
Mathematics achievement	−0.11 (0.08)	−0.11 (0.07)	0.08 (0.12)	−0.22 (0.17)

*Note*. Means with different subscripts in the same row are significantly different at *p* < 0.05. Values in parentheses are standard errors

Among the four groups of girls, a test of overall mean differences was marginally significant for English achievement, *χ*^2^(3) = 6.53, *p* = 0.09. Pairwise comparisons indicated that relational girls outperformed all other girls in English. The four groups of girls, however, did not differ significantly from each other on mathematics achievement, *χ*^2^(3) = 2.78, *p* = 0.43^1^. This was somewhat surprising given that the four profiles displayed distinct patterns of motivation and engagement in mathematics.

This study replicated mean-level differences in motivation, engagement, and achievement between boys and girls. However, by focusing on variations within gender, this study further shows that two thirds of the boys were motivated, engaged, and performed well in school. In contrast, while girls as a group are often considered diligent and high achieving students, the findings highlight the worrying patterns of motivation, engagement, and achievement among wild girls and modern girls.

## Discussion

Research on educational gender gaps has focused primarily on average gender differences in school motivation, engagement, and achievement. The nuanced findings from the present study illustrate the importance for quantitative researchers to move beyond a binary perspective and to pinpoint which boys and which girls are falling behind in school. Using latent profile analysis, the present study identified seven profiles of adolescents with similar patterns of gender role conformity and documented each profile’s prevalence. Further, these gender role profiles showed differential relations with students’ motivation, engagement, and achievement in English and mathematics. Within-gender variations indicate that two thirds of the boys were doing fine in school, while a sizable proportion of girls could be considered at risk. These results reveal the near invisibility of well-performing boys and underachieving girls in academic discourse.

### Subgroups of Adolescent Boys and Girls in School

Three groups of boys (resisters, cool guys, tough guys) and four groups of girls (relational girls, modern girls, tomboys, wild girls) emerged in the study, each showing a unique pattern of gender role conformity. These profiles map well onto existing images of boys and girls documented in prior studies, suggesting that the profiles identified here are likely to be robust.

Among boys, the current study identified a group of cool guys who behaved in a macho manner while placing importance on appearance and romance. Since physical dominance, attractiveness, and heterosexual success are robustly linked to boys’ popularity in adolescence (Rose et al. 2011), cool guys are likely to be a socially visible, high-status group in school. Adolescents similar to this profile have been widely studied under several different labels, notably the “lads” in the UK (Jackson 2006a) and the “jocks” in the US (Pascoe 2003). A profile consistent with the image of tough guys in previous studies was also found. In a study of adult men in the US, a tough guy identity was similarly associated with endorsement of emotional stoicism, extreme self-reliance, and physical aggression (Smiler 2006). Although both cool guys and tough guys in the current study displayed aggressive and macho behaviors, these two profiles could be distinguished by their differential endorsement of feminine norms. This finding speaks to the importance of examining young people’s adherence to both their own gender’s and the other gender’s norms to fully understand how they “do gender” in school.

Furthermore, the current study identified a group of boys who showed an inclusive form of masculinity and resisted the norms of emotional stoicism, competitiveness, violence, extreme self-reliance, and risk-taking. Although research has predominantly focused on boys and men who conform to conventions of masculinity, the current study showed that resistance to traditional masculinity was prevalent among adolescent boys (69%), and boys upholding traditional male gender norms were in the minority. This pattern is strikingly similar to the findings of a longitudinal qualitative study in the US (Way et al. 2014). By following a group of ethnically diverse boys from 6^th^ to 11^th^ grades, this study concluded that 71% of the boys resisted conventions of masculinity in early and mid-adolescence. Additionally, “average Joe”, “family man”, and “sensitive new man” were found to be the most frequently endorsed identities in a study of US adult men, and identification with these images was associated with nonadherence or resistance to traditional masculine norms (Smiler 2006). Taken together, findings across these diverse samples indicate that the prevalence of resistance to traditional masculinity may not be limited to a particular developmental stage or context. Despite the clear academic and psychological benefits associated with resistance to traditional masculinity during adolescence (A. A. Rogers, DeLay, et al. 2017), there is a lack of research into the factors that may support boys’ resistance to restrictive masculine norms (for an exception, see Way 2011). Future research should be careful in labeling boys and men who demonstrate nonconformity to gendered norms as subordinate or marginal (Paechter 2012), and instead examine what facilitates their healthy resistance to traditional masculinity.

Among girls, tomboys’ pattern of gender role conformity supports previous findings and suggests that a tomboy identity is characterized by simultaneously embracing masculinity while rejecting femininity (Paechter 2010). Wild girls similarly enacted stereotypically masculine behaviors but also invested heavily in an overtly feminine appearance and romantic relationships. Previous studies show that teachers and students in English schools can distinguish between tomboys and wild girls: while tomboys are viewed as one of the boys, wild girls are portrayed as wearing excessive makeup and tight clothing and being attractive to boys (Jackson 2006b). Since physical appearance and romantic success are closely tied to girls’ popularity during adolescence (Adler et al. 1992), wild girls are likely to have a high social standing in school. In addition, the current study found a group of relational girls who rejected the majority of gendered norms and showed the opposite pattern of gender role conformity to wild girls. Not only did relational girls shun competitiveness and aggression but they also rejected the thin body ideal that was highly valued among wild girls. This is consistent with the findings of a recent qualitative study (Paechter and Clark 2016), which similarly found that some British schoolgirls positioned themselves in opposition to the “cool girls” in school.

Finally, nearly half of the girls were classified as modern girls. Similar to wild girls, the modern girl profile was characterized by a juxtaposition of masculinity and femininity but in a less extreme manner. In a recent study, adolescent girls claimed that “we’re supposed to look like girls, but act like boys” (L. O. Rogers et al. 2020). Echoing this sentiment, modern girls in the current study subscribed to conventional ideals of feminine beauty, while striving for an appearance of strength by keeping problems to themselves and disconnecting from others emotionally. Given the crucial role of interpersonal connection in human thriving (Baumeister and Leary 1995), this pattern of gender role conformity is likely to engender tensions for modern girls (and wild girls): they might be simultaneously constrained by the restrictive norms about feminine appearance while unable to exercise the feminine strength of building connections with others.

The quantitatively derived profiles map well onto existing images of schoolboys and schoolgirls in the literature. This provides some validity evidence for the seven profiles and enhances the generalisability of masculinity and femininity typologies developed in small-scale research. Additionally, the current study reveals the relative size of each profile in a large sample of English secondary students, and suggests that prior studies may have focused on a small subset of young people who are socially visible while overlooking the voice and experience of those in the majority.

### Which Boys and Which Girls Are Falling Behind in School?

The current study demonstrates that adolescents’ patterns of conformity to a range of masculine and feminine norms can work in tandem to shape their academic success. Boys and girls who rigidly adhered to gender norms were less academically successful than those who showed resistance across gender norms. This result is consistent with prior studies showing the academic costs of strict adherence to traditional gender expectations (Ueno and McWilliams 2010).

Among boys, cool guys—who strongly endorsed all masculine norms—reported low perseverance and heightened self-handicapping, as well as performed the worst in English and mathematics. Previous studies indicate that rigid enactment of traditional masculinity can undermine boys’ achievement by reducing their likelihood of seeking help in academic contexts (Kessels and Steinmayr 2013). The current study points to a lack of perseverance and heightened self-handicapping as additional pathways through which traditional masculinity affects boys’ achievement. These maladaptive behaviors among cool guys might be in part explained by their strict adherence to winning and risk-taking. The mere thought of putting forth effort and failing might be sufficient to prompt these boys to adopt the risky strategy of self-handicapping. In this way, they can preserve the illusion that they can win and outperform others if they try. Tough guys, on the other hand, did not endorse the norms of winning and risk-taking and were much less likely than cool guys to self-handicap. Lastly, the largest profile of boys, namely resisters, reported a growth mindset and willingness to persevere with schoolwork and were performing well in English and mathematics. These variations in motivation, engagement, and achievement across the three groups challenge the simplistic framing of the “underachieving boys” debate and paint a more accurate picture of boys’ problems in education.

Although girls on average outperform boys in secondary school (Voyer and Voyer 2014), findings from the current study highlight the continuing disadvantage of some girls. Wild girls and modern girls—who made up half of the girls in this study—could be considered academically at risk: they reported a fixed mindset, low perseverance, and heightened self-handicapping in English and mathematics. A recent study revealed that girls had an increased tendency to give up and self-handicap after the transition to secondary school (Burns et al. 2019). Findings from the current study suggest that the growing disengagement among girls might be driven by wild girls and modern girls. In contrast, the female advantage in school might be primarily attributed to relational girls. These girls exhibited the most adaptive patterns of motivation and engagement across both subjects, and considerably outperformed other girls in English. Compared to other groups of girls, relational girls firmly rejected physical aggression and risky behaviors. As a result, they might experience more positive relationships with their teachers and peers, which could protect them against the decline in motivation and engagement in secondary schools (Burns et al. 2019).

The four groups of girls, however, did not differ significantly in their mathematics achievement. This is the case even though the four groups varied in their gender role profiles as well as patterns of motivation and engagement. The finding aligns with previous studies showing that adolescent girls’ degree of gender role conformity was unrelated to their mathematics performance (Yavorsky and Buchmann 2019). This suggests that some other factors, such as gender stereotypes or gender differences in self-efficacy, might suppress girls’ mathematics achievement across the board (Plante et al. 2013). Future research could investigate multiple factors known to inhibit girls’ mathematics performance and evaluate their relative contributions to the gender gap. This knowledge is useful for fine-tuning interventions designed to ameliorate gender disparities in mathematics.

Existing studies on within-gender variability in achievement often rely on male-only or female-only samples and provide gender-specific explanations as to why some boys or girls perform less well academically. By studying both genders together and assessing their conformity to both masculine and feminine norms, the current study suggests two general mechanisms through which gender role adherence might undermine boys’ and girls’ achievement. First, strict adherence to traditional gender roles can interfere with boys’ and girls’ academic success when the task or domain is experienced as incongruent with their gender roles (Elmore and Oyserman 2012). Among the seven profiles identified in this study, tough guys and tomboy girls adhered to masculine norms and rejected feminine norms. These two groups also performed well in mathematics but not in English, suggesting that doing well in a female-typed subject might be viewed as incompatible with their gender roles. In contrast, resister boys and relational girls rejected rigid constructions of gender, and this gender role expansion was associated with positive academic adjustment. These two groups were willing to display effort and engagement even in subjects that could be viewed as counter-stereotypical to their gender.

Second, young people who adhere to gendered ideals of behavior and appearance might place a high value on peer status and, therefore, experience greater conflict between maintaining peer status and trying hard in school. Cool guys, modern girls, and wild girls attached importance to gender-normative behaviors, attractive appearance, and romantic relationships—factors that have been linked to increased popularity during adolescence (Mayeux and Kleiser 2019). Meanwhile, academic effort is perceived as uncool during adolescence, and adolescent boys and girls displaying high effort are rated by their peers as lower in popularity (Heyder and Kessels 2017). Given this conflict between school effort and peer status, young people with the desire to gain or maintain peer approval tend to purposely withhold effort in school (Yu and McLellan 2019). Indeed, cool guys, modern girls, and wild girls in the current study reported low perseverance and frequent use of effort withdrawal as a self-handicapping strategy.

Findings from the present study challenge the practice of treating boys and girls as two uniform groups in gender gap research. The findings further suggest that explanations that have been traditionally used for boys’ underachievement, including (a) the incompatibility between gender roles and the image of certain subjects and (b) the conflict between schoolwork and popularity, might apply to both genders.

### Implications for Practice

Given the academic costs associated with rigid adherence to traditional gender norms and the benefits associated with resistance, fostering resistance to traditional masculinity and femininity may reduce the gender role conflict experienced by some young people and increase their school engagement and achievement. A recent study found that even when young men rejected traditional masculine norms privately, they felt pressure to conform to these norms because they overestimated their peers’ support for such norms (Van Grootel et al. 2018). However, as discussed earlier, findings from the current research and several other studies indicate that resistance to masculine ideals may be the rule rather than the exception. Highlighting the prevalence of resistance can debunk some students’ false beliefs and allow them to act more in line with the real norm and their true selves. By presenting individuals with accurate information about their peers and highlighting the discrepancy between the perceived and actual norms, brief social norms interventions have successfully helped young men to feel more comfortable about expressing their feelings (Beatty et al. 2007) and hold more egalitarian beliefs about gender (Kilmartin et al. 2008).

Furthermore, young people’s peer relationships provide key developmental contexts that shape their gender role attitudes (Kågesten et al. 2016). Although peer groups can create pressure for gender role conformity (Adler et al. 1992), reliable and trusting friendships can provide young people with a safe space to challenge traditional gender norms. Studies show that boys with close male friendships are more likely to maintain their resistance to emotional stoicism, physical aggression, and extreme self-reliance (Way 2011). Likewise, girls who are secure and confident in their friendships tend to be less concerned about striving for feminine beauty, romance, or popularity (Gulbrandsen 2003). Cultivating positive and trusting friendships in adolescence may therefore provide young people with the necessary social capital to resist gender norms.

### Limitations and Future Directions

This study has several limitations that could be addressed in future research. Although this study utilized a large sample drawn from four different schools, the generalisability of the profiles as well as the relationship between the profiles and academic outcomes warrant additional investigation. The current study focused on adolescents’ patterns of conformity to nine dominant norms, and future studies could broaden the scope to include other salient gender norms. For example, being obedient and agreeable are often viewed as important for the construction of femininity. The addition of other norms could change the final solution of the profiles as well as the relationship between the profiles and outcome variables. However, it is possible that the level of conformity similarly matters for other gendered norms. Although being obedient and agreeable may be seen as positive in the school context, strict and rigid adherence to these norms can become problematic when they result in submissiveness and self-silencing.

Although the analyses focused on within-gender variations in gender role conformity and academic outcomes, the current study provides some clues as to how social class and race/ethnicity might shape adolescents’ construction of gender. Due to the small number of participants in each ethnic group, they were aggregated into one category and were contrasted with White students in analyses. However, this practice masks the heterogeneity among people from diverse ethnic and cultural backgrounds. Future studies could extend the current study with larger and more diverse samples and examine whether students of different minority backgrounds are differentially represented in the obtained profiles. In addition, gender role measures used in this study are designed to assess conformity to gendered norms rooted in the dominant (i.e., White) culture in the US and may not adequately capture the conceptions of masculinity and femininity among different ethnic groups. Future studies should continue to investigate the construction of gender from an intersectional lens and include more culturally relevant gender norms.

From a developmental perspective, there may be age-related changes in how people construct their masculinity or femininity. Even when similar profiles emerge in other studies, the size of these profiles is likely to differ across developmental stages. For example, research suggests that many girls cease to be tomboys when they enter adolescence (Carr 2007). As a result, a longitudinal study that identifies gender role profiles across multiple time points could reveal interesting changes in people’s patterns of gender role conformity. Lastly, although connections have been made between the obtained profiles in this study and existing images of boys and girls in the literature, these links are tentative. Future research would benefit from adopting a mixed-method approach and conducting follow-up interviews with prototypical members of each profile. Data generated from this qualitative phase can provide a richer understanding of how young people accommodate or resist traditional gender expectations.

## Conclusion

The majority of research on gender gaps in school focuses on average differences between genders, rendering many well-performing boys and low-achieving girls invisible. To unpack the vast variability within each gender, the present study quantitatively mapped out the different ways adolescents enacted their gender and pinpointed which boys and girls were most at risk academically. Three groups of boys (resisters, cool guys, tough guys) and four groups of girls (relational girls, modern girls, tomboys, wild girls) were identified. Within-gender variations show that half of the boys were doing fine in school, while half of the girls displayed worrying patterns of motivation, engagement, and achievement. These findings illustrate the importance of adopting a “which boys and which girls” approach in educational gender gap research.

## Supplementary information

Supplementary Materials

## Footnotes

1 Since socioeconomic status and race/ethnicity were related to girls’ profile membership, main analyses were rerun to include them as additional covariates. This did not change the pattern of group differences in English and mathematics achievement.
