# Supplementary material for: Which Boys and Which Girls Are Falling Behind? Linking Adolescents’ Gender Role Profiles to Motivation, Engagement, and Achievement
Source: J Youth Adolesc. 2020 Jul 31;50(2):336–52. doi: 10.1007/s10964-020-01293-z (PMC7875942; doi:10.1007/s10964-020-01293-z)
Supplement: Supplementary file 1 — Supplementary Materials [file 10964_2020_1293_MOESM1_ESM.docx]

**Supplementary Materials**

**Supplementary Appendix A.** Fit indices for the measurement invariance models

| Model | $\chi^{2}$ | *df* | CFI | RMSEA | SRMR | $\Delta$CFI | $\Delta$RMSEA | $\Delta$SRMR |
| --- | --- | --- | --- | --- | --- | --- | --- | --- |
| 1 Configural | 2252.96 | 1464 | .962 | .042 | .031 |  |  |  |
| 2 Weak | 2432.74 | 1815 | .970 | .034 | .043 | .008 | –.008 | .012 |
| 3 Strong | 2560.27 | 1902 | .968 | .034 | .045 | –.002 | .000 | .002 |
| 4 Strict | 2674.69 | 1950 | .965 | .035 | .046 | –.003 | .001 | .001 |

**Supplementary Appendix B.** Fit indices for latent profile analyses

| Profile | LL | #fp | AIC | CAIC | BIC | SABIC | *p*BLRT | Entropy |
| --- | --- | --- | --- | --- | --- | --- | --- | --- |
| Boys |  |  |  |  |  |  |  |  |
| 2 | -3823.50 | 28 | 7703.00 | 7835.26 | 7807.26 | 7718.45 | <.001 | .77 |
| **3** | **-3779.18** | **38** | **7634.35** | **7813.85** | **7775.85** | **7655.33** | **<.001** | **.81** |
| 4 | -3753.39 | 48 | 7602.77 | 7829.51 | 7781.51 | 7629.27 | <.001 | .81 |
| 5 | -3730.84 | 58 | 7577.68 | 7851.65 | 7793.65 | 7609.70 | <.001 | .81 |
| 6 | -3708.86 | 68 | 7553.72 | 7874.93 | 7806.93 | 7591.26 | <.001 | .83 |
| Girls |  |  |  |  |  |  |  |  |
| 2 | -3632.20 | 28 | 7320.40 | 7451.25 | 7423.25 | 7334.46 | <.001 | .61 |
| 3 | -3579.40 | 38 | 7234.80 | 7412.38 | 7374.38 | 7253.88 | <.001 | .67 |
| **4** | **-3545.84** | **48** | **7187.68** | **7411.99** | **7363.99** | **7211.78** | **<.001** | **.76** |
| 5 | -3513.67 | 58 | 7143.33 | 7414.38 | 7356.38 | 7172.45 | <.001 | .79 |
| 6 | -3486.28 | 68 | 7108.55 | 7426.34 | 7358.34 | 7142.70 | <.001 | .77 |

**Supplementary Appendix C.** Elbow plots for latent profile analyses

**Supplementary Appendix D.** Average latent profile probabilities (row) for most likely profile membership (column)

Boys

| Most likely profile membership | Resisters | Cool guys | Tough Guys |
| --- | --- | --- | --- |
| 1 | **.94** | .05 | .02 |
| 2 | .09 | **.88** | .03 |
| 3 | .10 | .05 | **.86** |

Girls

| Most likely profile membership | Relational girls | Modern girls | Tomboys | Wild girls |
| --- | --- | --- | --- | --- |
| 1 | **.87** | .11 | .03 | .00 |
| 2 | .09 | **.87** | .03 | .02 |
| 3 | .05 | .09 | **.86** | .00 |
| 4 | .00 | .11 | .00 | **.89** |

**Supplementary Appendix E.** Mean values of profile indicators

Boys

| Variable | 1 Resisters | 2 Cool Guys | 3 Tough Guys |
| --- | --- | --- | --- |
| Emotional control | -0.39_b_ | 0.55_a_ | 1.33_a_ |
| Winning | -0.26_b_ | 1.05_a_ | -0.48_b_ |
| Violence | -0.40_b_ | 1.05_a_ | 0.38_a_ |
| Self-reliance | -0.22_b_ | 0.32_a_ | 0.75_a_ |
| Risk-taking | -0.25_b_ | 0.91_a_ | -0.25_b_ |
| Thinness | 0.04 | -0.10 | -0.02 |
| Appearance orientation | 0.05_a_ | 0.36_a_ | -1.07_b_ |
| Romantic relationship | 0.10_a_ | 0.28_a_ | -1.19_b_ |
| Domestic | 0.13_a_ | -0.14_ab_ | -0.54_b_ |

*Note*. Numbers that do not share a letter are significantly different at *p* < .05.

Girls

| Variable | 4 Relational Girls | 5 Modern Girls | 6 Tomboys | 7 Wild Girls |
| --- | --- | --- | --- | --- |
| Emotional control | -0.82_b_ | 0.33_a_ | 0.66_a_ | 0.32_a_ |
| Winning | -0.32_b_ | 0.04_a_ | 0.43_a_ | 0.42_a_ |
| Violence | -0.49_b_ | 0.16_a_ | 0.32_a_ | 0.63_a_ |
| Self-reliance | -0.78_b_ | 0.37_a_ | 0.22_a_ | 0.68_a_ |
| Risk-taking | -0.52_b_ | 0.20_a_ | 0.19_a_ | 0.72_a_ |
| Thinness | -0.49_c_ | 0.31_b_ | -0.80_c_ | 1.66_a_ |
| Appearance orientation | -0.10_b_ | 0.19_b_ | -1.16_c_ | 1.33_a_ |
| Romantic relationship | 0.01_b_ | 0.14_b_ | -1.46_c_ | 1.77_a_ |
| Domestic | 0.41_a_ | -0.22_b_ | 0.00_ab_ | -0.43_b_ |

*Note*. Numbers that do not share a letter are significant different at *p* < .05.

**Supplementary Appendix F.** Multinomial logistic regressions predicting profile membership

Boys

|  | 2 vs. 1 | | 3 vs. 1 | | 3 vs. 2 | |
| --- | --- | --- | --- | --- | --- | --- |
| Predictor | Coef. | *OR* | Coef. | *OR* | Coef. | *OR* |
| FSM | -0.84 | 0.43 | -1.78 | 0.17 | -0.94 | 0.39 |
| Non-White | 0.75 | 2.12 | -1.31 | 0.27 | -2.06 | 0.13 |

*Note*. Coef. = coefficient; *OR* = odds ratio. The coefficients and *OR*s reflect the effects of predictors on the likelihood of membership into the first listed profile relative to the second listed profile. Profile 1 = Resisters; Profile 2 = Cool Guys; Profile 3 = Tough Guys. FSM = free school meal status.

Girls

|  | 5 vs. 4 | | 6 vs. 4 | | 7 vs. 4 | | 6 vs. 5 | | 7 vs. 5 | | 7 vs. 6 | |
| --- | --- | --- | --- | --- | --- | --- | --- | --- | --- | --- | --- | --- |
| Predictor | Coef. | *OR* | Coef. | *OR* | Coef. | *OR* | Coef. | *OR* | Coef. | *OR* | Coef. | *OR* |
| FSM | 1.69* | 5.40 | 1.91* | 6.75 | 1.31 | 3.71 | 0.22 | 1.25 | -0.38 | 0.69 | -0.60 | 0.55 |
| Non-White | -0.48 | 0.62 | 0.77 | 2.15 | -1.51 | 0.22 | 1.25** | 3.48 | -1.03 | 0.36 | -2.28 | 0.10 |

*Note*. Coef. = coefficient; *OR* = odds ratio. The coefficients and *OR*s reflect the effects of predictors on the likelihood of membership into the first listed profile relative to the second listed profile. Profile 4 = Relational Girls; Profile 5 = Modern Girls; Profile 6 = Tomboys; Profile 7 = Wild Girls. FSM = free school meal status.

* *p* < .05, ** *p* < .01.

**Supplementary Appendix G.** Differences in mindset, perseverance, and self-handicapping across profiles

Boys

| Variable | 1 Resisters | 2 Cool Guys | 3 Tough Guys |
| --- | --- | --- | --- |
| English mindset | .09_a_ | -.36_b_ | -.03_ab_ |
| English perseverance | .13_a_ | -.63_b_ | -.48_b_ |
| English self-handicapping | -.07_b_ | .51_a_ | -.28_b_ |
| Math mindset | .20 | .15 | -.13 |
| Math perseverance | .18_a_ | -.18_b_ | -.25_b_ |
| Math self-handicapping | -.11_b_ | .30_a_ | -.19_b_ |

*Note*. Numbers that do not share a letter are significantly different at *p* < .05.

Girls

| Variable | 4 Relational Girls | 5 Modern Girls | 6 Tomboys | 7 Wild Girls |
| --- | --- | --- | --- | --- |
| English mindset | .33_a_ | -.14_b_ | -.05_b_ | -.21_b_ |
| English perseverance | .54_a_ | -.05_b_ | -.09_b_ | -.48_b_ |
| English self-handicapping | -.40_b_ | .20_a_ | -.35_b_ | .61_a_ |
| Math mindset | .29_a_ | -.45_b_ | -.28_b_ | -.07_ab_ |
| Math perseverance | .41_a_ | -.28_b_ | -.27_b_ | -.38_b_ |
| Math self-handicapping | -.42_b_ | .33_a_ | -.29_b_ | .71_a_ |

*Note*. Numbers that do not share a letter are significant different at *p* < .05.
